# Peroral endoscopic myotomy for esophageal motility disorders

**DOI:** 10.1007/s10388-019-00693-w

**Published:** 2019-10-12

**Authors:** Jie Feng, Raja Waqar Ali, Jin-yong Hao, Gui-xiang Kong, Li-hong Yang, Xiao-jun Huang

**Affiliations:** 1 Department of Gastroenterology, Lanzhou University Second Hospital, NO. 82 Cuiying Men, Cheng Guan District, Lanzhou, 730030 Gansu Province China; 2 Nationality: Pakistan, Lanzhou University Second Hospital, Lanzhou, 730030 Gansu Province China

**Keywords:** Peroral endoscopic myotomy, Achalasia, Esophageal motility disorders

## Abstract

**Background:**

Esophageal motility disorders which include achalasia, esophagogastric junction outflow obstruction (EGJ outflow obstruction), jackhammer esophagus (JE), distal esophageal spasm (DES), etc. are rare disease of unknown causes. The diagnosis is based on endoscopy, barium meal, and high-resolution manometry (HRM). With the development of endoscopy, peroral endoscopic myotomy (POEM) has emerged as a standard method for the treatment of achalasia.

**Purpose:**

The purpose of this article is to enable gastroenterologists to have a more comprehensive understanding of the application status, technical characteristics, clinical efficacy and future prospect of POEM in the treatment of esophageal motility disorders.

**Methods:**

Through a large number of reading literature, combined with clinical practice, summary and analysis of the indications, procedure, efficacy, complications, and controversies of POEM in the treatment of esophageal motility disorders, as well as the current and future perspectives of POEM were studied.

**Results:**

POEM is safe and effective in the treatment of esophageal motility disorders, but the GERD reflux rate is higher.

**Conclusions:**

POEM can be a new option for the treatment of esophageal movement disorders, but large sample, multi-center, long-term study reports are needed, and it promotes the development of NOTES technology.

## Introduction

**Fig. 1:**
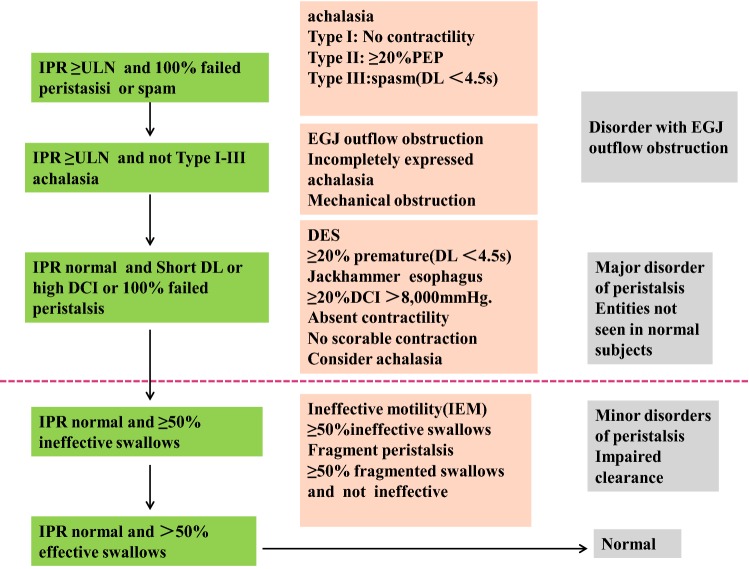
Hierarchical algorithm of Chicago classification v.3.0. *IPR* Integrated relaxation pressure, *DCI* distal contractile integral, *ULN* upper limit of normal, *DL* distal latency, *PEP* panesophageal pressurizations, *IEM* ineffective motility

High-resolution manometry (HRM) is now considered as the gold standard diagnostic tool [1], which has four major categories that are classified on the basis of lower esophageal sphincter (LES) relaxation and motility of esophageal body: (1) incomplete LES relaxation, including achalasia and EGJ outflow obstruction; (2) major motility disorders, including absent contrility, DES, hypercontrile and JE; (3) minor motility disorders, including ineffective esophageal motility (IEM) and fragment edperistalsis; (4) normal esophageal motility [2] (Fig. 1). The appropriate intraoperative HRM diagnosis determines the choice of treatment method and can predict the management effect.

Treatment of esophagus motility disorders includes drugs, laparoscopic Heller myotomy, endoscopic botox injections, and pneumatic dilation (PD). Laparoscopic Heller myotomy has been the widely used treatment for the achalasia. In 1980, Ortega et al. [3] first reported the treatment of achalasia by endoscopic lower esophageal sphincterotomy, which has not been widely used due to poor operability and clinical efficacy. In 2007, Sumiyama et al. [4] discussed the technical feasibility and safety of applying submucosal skin flap for total gastrectomy into the abdominal cavity in pigs. In 2009, Inoue et al. [5] first tried a new endoscopic technique for the treatment of achalasia, which was named POEM. To our knowledge, there are approximately 10,000 cases performed worldwide and the number is increasing exponentially. POEM has become the standard treatment of achalasia and gradually introduced to the treatment of non-achalasia esophageal motility disorders [6, 7].

### Indications and contraindications

#### Indications

The application of early POEM mainly relieves the clinical symptoms of non-sigmoid colon achalasia. With the study of safety and effectiveness of the clinical reports POEM has also been introduced to the treatment of sigmoid colon achalasia. Recently, the feasibility of POEM has been shown not only in primary idiopathic achalasia, but also in other particular patient cohorts. Comparative indication for POEM includes patients with special achalasia, recurrent achalasia subsequently prior treatment failure (PTF) and non-achalasia esophageal motility disorders as well as gastroparesis have been studied (Table 1).

**Table 1:** Indications of POEM

Diseases	Classifications
Achalasia	Type I–III achalasia
Special achalasia	Sigmoid achalasia
	Pediatric achalasia [10–13]
	Achalasia with Roux-en-y gastric bypass previous [14]
	Achalasia with diverticulum [15]
Recurrent achalasia	Failed Heller myotomy
	Failed pneumatic balloon dilatation
	Failed previous POEM
	Falied previous botox injections
Non-achalasia esophageal motility disorders	JE/Nutcracker esophagus(NE)
	EGJ outflow obstruction
	DES
Zenker’s diverticulum [16] and Gastroparesis	

#### Contraindications

POEM is absolutely contraindicated mainly with other serious diseases that cannot be implemented with general anesthesia and tracheal intubation patients [8] and with those who have intervention in esophageal such as a large area of endoscopic mucosal resection (EMR) in esophagus, endoscopic mucosal dissection (ESD), radiofrequency ablation, radiation therapy [9], as well as a large ulcer in the lower esophagus. However, studies [8] have showed that preoperative severe gastroesophageal reflux as a relative contraindication of POEM suggesting that those patients should have laparoscopic Heller myotomy and fundoplication to reduce the incidence of reflux.

## The procedure of POEM

Preoperative preparation is an important step of POEM. Scholars suggest a fluid diet for 48 h [8]. However, the duration of fasting varies slightly from center to center. The differences may be related to the severity and achalasia type of patients in each center. The more severe the disease, the more strict the preoperative fasting is required. In our center, 48 h is recommended before operation. On the other hand, administration within 24 h before operation could induce the esophageal cavity negative pressure in patients and suction pressure to cleaning up the esophagus wall. Prior to POEM, it requires that the esophagus be rinsed endoscopically to ensure that the esophageal contents are cleared. There is no consensus on whether the antibiotic solution should be added during cleaning [8, 17].

### Mucosal incision and tunnel entry

Most of the recent researches on POEM are based on the classic operation procedures as reported by Inoue [5]. Initially, for simple achalasia patients, HRM is limited to incomplete relaxation in LES and generally, the submucosal injection site is usually 10 cm away from the EGJ oral cavity end. However, for non-achalasia motility disorders, HRM is mainly manifested in esophageal body motility disorder. So, it is necessary to determine the beginning and termination of abnormal contraction according to HRM results. According to HRM results, the position of mucosal incision in the operation should be 2 cm higher than the initial end of abnormal contraction. The anterior, posterior or right side wall of the esophagus was selected as the injection point which also determined the location of the submucosal incision, the direction of tunnel establishment and the site of myotomy. After lifting of the mucosa, a longitudinal incision is made with a diameter of about 2 cm. Inoue et al. [18] suggested that the tunnel should be located in the anterior or posterior wall. Wang et al. [19] demonstrated that simple longitudinal mucosal incision tunnel width ≤ 3 cm and sigmoid-type esophagus are independent risk factors for gas-related complications for achalasia during POEM. Ma and colleagues [20] established a T-shaped incision and they found the T-shaped incision is more favorable to endoscopic entry thus reducing the pressure in the tunnel and facilitating liquid discharge in the tunnel. However, there is no prospective and large sample reported about the efficacy of T-shaped incision vs. longitudinal incision. The construction of a sigmoid achalasia tunnel should avoid the deviation of tunnel direction when the tunnel is near the EGJ, space is relatively narrow, while the vision is suddenly broadened when it reaches the gastric side. At this time, the endoscope should exit the tunnel and should observe whether the tunnel reaches 2–3 cm on the gastric side. For beginners, it is very necessary to timely exit the endoscopic lens from the tunnel cavity and enter the esophageal cavity for observation, which can help to establish a straight tunnel and myotomy in liner. In addition, the methylene blue into the injection and Endolumenal Functional Lumen Imaging Probe system (EndoFLIP) are conducive to the observation of the location of the tunnel. It is helpful to visualize the dissecting scope’s light with a second scope in the stomach.

### Myotomy

#### Circular myotomy or full-thickness myotomy?

Classic myotomy only separates the circular fibers of the esophagus, LES and stomach maintaining the integrity of the longitudinal muscular layer but few attempts have been made to dissect the entire muscular layer including the intrinsic muscle [21]. A study by Wang et al. [22] compares 32 circular myotomy to 24 full-thickness myotomy with complete gastroesophageal reflux disease (GERD) evaluation, they found the efficacy is comparable, whereas the patients who with full-thickness myotomy has low postoperative 4-s IRP as well as more GERD. The most likely reason is that the longitudinal muscles theoretically maintain the integrity of the anatomical structure and ensure a certain degree of anti-reflux. For non-achalasia motility disorder, there is no unified conclusion whether the circular myotomy or full-layer myotomy should be cut, because few reports have illustrated the issue.

#### The lengthen of myotomy

The length of myotomy should be individualized based on intraoperative endoscopic identification of the high-pressure zone as well as comparisons to the preoperative HRM topography and contrast studies, base on HRM, we have come to conceptualize esophageal motility disorders as characterized by obstructive physiology at the esophagogastric junction, smooth muscle esophagus, or both [23]. For achalasia, the common myotomy length average is 8–10 cm, However, different types of achalasia have different lengths of myotomy, the length of myotomy often needs to be longer than usual in cases of Chicago classification type III achalasia, DES, or JE [18]. Khashab and collages [24] analyzed 73 cases of patients with esophageal motility disorders from 11 centers: 9 cases of DES, 10 JE, and 54 cases of spastic achalasia, the mean length of myotomy was 16 cm, followed-up for 234 days, and 93% of the patients showed clinical remission, he also retrospectively analyzed 50 cases from 11 centers in 2018 [25], including 18 cases of JE, 17 DES, and 15 cases of EGJ outflow obstruction, the average length of myotomy was 15.1 ± 4.7 cm and which was significantly longer than that in achalasia patients. There’s a contradiction here, since some patients in HRM classification are just esophagus body movement disorders and do not have significant EGJ outflow obstruction [2], do we need to cut open LES in POEM? Unfortunately, this debate cannot be confirmed by the present study, as almost all studies have performed an incision of the LES. Bechara and colleagues [26] reported four cases of JE treated with POEM, three of whom had LES myotomy while the other one did not, patients incisioned with the LES had resolution in symptoms, whereas the latter one developed significant dysphagia and regurgitation, when the patient retreated again under POEM and the symptoms are alleviated after LES was cut open. It seems to have better effectiveness to cut open the LES as the LES pressure may result in postoperative dysphagia caused by induced aperistalsis. Nakato et al. [27] suggested that the patients should be followed up for at least 2 months before operation because of the variability of JE symptoms. Inoue [18] recommend the starting point of the myotomy is the oral side of the abnormal luminal obstructive contractions in the esophageal body and the endpoint is 1–2 cm into the gastric side to secure the LES incision. When approaching LES, the muscle layer becomes thicker and the tunnel cavity becomes narrow, so it is difficult to cut open. The application of transparent cap is helpful to broaden the field of vision. In addition, ensuring adequate intraoperative linear incision of LES is the key to POEM’s long-term efficacy when myotomy was done. It should be observed whether EGJ is relaxed than before or not. EndoFLIP is used to measure LES distensibility during the POEM to improve long-term outcomes by giving an intraprocedural indicator of the adequacy of the myotomy prior to tunnel closure [28].

#### The position of myotomy

Currently, a total of 3 myotomy sites have been reported during POEM for achalasia: anterior myotomy (12–2 o’clock orientation), posterior myotomy (5–6 o’clock orientation), and left posterior myotomy (8 o’clock orientation) [29]. Inoue et al. [18] recommended that the position of myotomy is in the anterior or posterior wall just as the way of the tunnel and the mucosal incision. However, Bechara et al. [30] have the view that posterior wall may be better than anterior wall no matter from the theory aspect, or from the practice aspect. But both of them consistently did not recommend the right side wall as the muscle strength of the right wall is weak, diverticulum may occur after the POEM. There is still no evidence yet has demonstrated which site is better.

### Closed tunnel entrance

After the completion of myotomy, ensure that there is no bleeding in the tunnel cavity, no liquid residue, and the damaged vessels in the process of electrocoagulation should be treated before exiting the tunnel, observed the integrity of the mucosa and then clamping tunnel opening from the anal side to the oral side with the metal clip.

## Clinical efficacy

### Efficacy of achalasia

Postoperative symptomatic score of patients with Eckardt was less than or equal to three points, and LES pressure was lower than that before surgery (the descending amplitude was greater than 50%), barium meal examination showed that improvement of emptiness before surgery was considered as treatment successfully. A single-center study [31] comprising of a large number of samples showed that 500 achalasia patients who successfully underwent POEM had a strictly limited Eckardt score of 2, and a postoperative effective rate was 91% at 6 months, and 3 years later, the effective rate was 88.5%. Akintoye et al. [32] conducted a meta-analysis of 2373 cases from 36 institutions in 12 countries, it was confirmed that the short-term effective rate was 98%. Since the successful operation of the first POEM was carried out for almost 10 years, the efficacy and safety of POEM to achalasia is indisputable. Its short-term curative effect can reach 90–100% [33, 34], the longest follow-up report details a success rate at 5 years of 83% [35]. Compared to standard Heller myotomy and PD the POEM has obvious advantages. A meta-analysis of 5834 patients undergoing POEM vs 1958 patients who undergoing Heller myotomy showed that the clinical symptom remission rates at 12 months and 24 months after POEM were higher than that of Heller myotomy (12 months: 93.55 vs. 91%, *p* = 0.01, 2 years: 92.7% vs. 90.0%, *p* = 0.01) [36]. A study by Meng et al. [37] compared patients who underwent POEM vs PD, and found that both groups faired similarly at 3 months (96% success for POEM vs 95% success for PD). However, at 3 years, the continued clinical success of PD dropped to 60%, whereas patients who received POEM maintained a 93% remission of symptoms. Compared with POEM’s stunning short-term effect, the long-term success rate will be reduced. Some patients suffer from recurrence of symptoms, which Li and his colleagues found an incomplete myotomy (i.e. insufficient incision below the EGJ) is the most likely explanation for persistent or early recurrent dysphagia [38]. Multivariate Cox regression analysis revealed long disease duration (≥ 10 years) and a history of prior interventions to be a risk for recurrence [39]. Nabi et al. [40] compared the patients who underwent POEM directly vs. the patients undergoing POEM after prior treatment failure (PTF), the clinical success rate was no obvious difference (92.4% vs 92.5% *p* = 0.95), whereas PTF patients showed significantly longer operation time. In addition, multi-factor analysis showed loss of flaccidity syndrome types, esophageal expansion (> 6 cm), the course of the disease, treatment methods, and the occurrence of adverse events and use of knife type is an important predictor of operation time.

### Efficacy of non-achalasia esophageal motility disorders (Table 2)

DES is mainly the muscular layer thickening in the lower 2/3 of the esophagus. GERD may be the early symptoms of DES, while in the later stage, DES may develop into achalasia. EGJ outflow obstruction is thought to be an early stage of achalasia, with at least some patients developing achalasia. Therefore, for non-achalasia esophageal motility disorders, correctly diagnosis as well as a certain follow-up time before operation was crucial. In 2012 and 2013, American and Japanese scholars [6, 41] have successively taken POEM for treating patients with DES. POEM is gradually becoming a new therapy of non-achalasia esophageal motility disorders. It can be seen from the Table 2 that the research focuses on JE, NE, DES and EGJ outflow obstruction, the clinical effective rate was 70–100%, most of the patients got clinical relief, the longest time of follow-up was 48 months, complication rates of 0–33%, the mean length of myotomy was from 9.9 ± 5.4 cm to 15.1 ± 4.7 cm, which was varies from patient to patient, most of this difference is due to individual differences in the disease itself. Although the current data show a better clinical remission rate, there are still some deficiencies, such as small sample size, lack of prospective and comparative studies. At present, there is no expert consensus or guidelines of POEM for the treatment of non-achalasia esophageal motility disorders.

**Table 2:** The efficacy of POEM for non-achalasia esophageal motility disorders

First authors (publication year)	Patients (*n*)	Mean Eckardt score		Mean mytomy length (cm)	Clinical responses (%)	Mean follow-up	Complications% (n/N)
		Pre-POEM	Post-POEM				
Louis [41]	1DES	7	1	13	100	2 M	0
Shiwaku [6]	1DES	7	0	17	100	NM	0
Kristens [42]	3NE	10,10,11	3,1,1	16	100	12 M	33.3
Khashab [24]	9DES	6.9	1	16	100	7.8 M	22.2
	10 JE	8.4	2.6		70		20
	54spastic achalasia	6.4	0.86		96.3		7.4
Sharata [43]	75achalasia	6	1	8	100	20.1 M	6
	25(12NE/5DES/8isolated hypertensive non-relaxing LES)	5			70	23.0 M	
Bechara [26]	4JE	5,5,11	6,0,0,2		75%	12 M	0
Khan [44]	37 JE	N	≤ 3	13.5	72%	N	16
	18DES				88%		14
Khashab [25]	15EGJ outflow obstruction (17DES/18JE)	6.2 6.9	1 1.9	15.1 ± 4.7	93.3% 84.9%	195 days	18
Filicori [45]	(15hypercontractile esophagus 11DES 14 EGJ outflow obstruction	5.02(± 0.27)	1.2	9.9 ± 5.4 7.4 ± 2.4 13.0 ± 6.2	91%	48 M	10

## Complications

POEM has a low incidence of perioperative complications and is mostly avoided or cured with conservative treatment. It should be operated by legally licensed medical centers. In addition, effective anesthesia and intensive care ensures a safe and effective procedure. Our experience is that operators should receive professional technical training, having more than 20–30 cases of esophageal ESD treatment experience and the experience in dealing with intraoperative bleeding, perforation and other complications. It should be done under the guidance of experienced physicians at the early stage. A report by Liu et al. [46] shows that operating 100 cases of POEM can reduce the incidence of complications and improve the success rate and completing 70 cases can significantly shorten the operation time. The learning curve of POEM has been addressed in multiple publications, but proper formal training guidelines have not been formulated yet. Detailed training programs are urgently needed. GERD remains a long-term problem. A study [31] reported by Inoue showed incidence of reflux esophagitis were observed after surgery upon 2 months, and 3 years as 16.8% and 21.3%, respectively. Hernandez et al. [47] analyzed 68 patients in the pH study, endoscopy, and questionnaire according to the follow-up time, the pH positive was over 55%, the endoscopy positive was over 28%, whereas the reflux symptoms was less than 15% at 12 months. Once the proton pump inhibitor administrated the percentages decreased to 3%, 1%, and 4% at 60 months, respectively. The application of PPI agents greatly controlled the symptoms of reflux. Compared to the Heller myotomy, the incidence of GERD is higher in most reports, although few reports suggest that there is no difference [34, 36]. In addition, POEM can lead to significant increase in abnormal esophageal acid exposure rate while the incidence of GERD symptoms do not [48] and it may be due to the reason that POEM does not combine with laparoscopic fundoplication. In order to overcome the higher incidence of GERD. Recently, a pilot study was conducted by Inoue et al. [49] in which an endoscopic fundoplication was added to the standard POEM (POEM  +  F) procedure. On follow-up endoscopy at 2 months, almost all patients visually appeared to maintain the wrap across the GEJ. Nevertheless, larger prospective studies are needed to evaluate the efficacy of technique of POEM + F which may help mitigate the post-POEM incidence of GERD.

### Current and future perspectives

Comparing with Heller myotomy and PD, POEM is minimally invasive and free of scars on the body surface and its short-term curative effect is equal to or better. In the meantime, it has more endurance and can be chosen at any length and direction of incision according to different conditions. Whereas POEM is currently in its tenth year of practice and its 5-year efficacy is similar to that of Heller myotomy, which is more than 100 years old. Furthermore, due to the lack of anti-reflux in POEM routine, current studies report that the incidence of postoperative reflux is much higher than that of Heller. Currently, there are no high-quality studies that directly compare the efficacy of POEM and Heller with that of gastroesophageal reflux. In the future, the research of endoscopic anti-reflux therapy will also be an urgent problem of POEM. Therefore, currently, Heller is the gold standard for the treatment of achalasia and it has been accepted by a large number of clinical practices setting for a long time. Pending to the long-term efficacy and gastroesophageal reflux data results are available POEM will replace Heller’s idea which is currently lacking of evidence.

Apart from the treatment of esophageal motility disorders, the tunneling technology based on POEM makes a great contribution to the development of minimally invasive treatment of digestive endoscopy. Submucosal tunneling endoscopic resection (STER) has been accepted by most digestive endoscopy centers as compared with the perforation risk caused by traditional endoscopic resection of submucosal tumors, STER provides a minimally invasive endoscopic treatment with intact mucosa and low risk of complications, especially for tumors originating from intrinsic muscle layer.

Gastric-POEM (G-POEM) is mainly used for the treatment of gastroparesis. Currently, there are approximately 200 cases of such patients reported worldwide. Khashab and colleagues [8] concised that the surgical success rate of G-POEM is 100% and the clinical success rate is 69–85%. Currently, there are no randomized, prospective or multicentre studies addressing this issue.

Although the concept of natural orifice transluminal endoscopic surgery (NOTES) has been put forward by Kalloo and Kantsevoy for more than 20 years, NOTES technology hasn't evolved greatly. While the POEM technology under the tunnel has greatly promoted the development of NOTES technology, long-term practice and development have enabled NOTES technology to overcome the unsatisfying period and move towards the stage of rapid development [50].

## Conclusion

POEM has gained popularity in the treatment of achalasia and other esophageal motility disorders worldwide and most of reports have demonstrated the remarkable efficiency of POEM. However, to maintain long-term efficacy and find an endoscopic anti-reflux therapy is still a challenge of POEM. Tunnel-based minimally invasive endoscopic treatment just as NOTES and STER has shown a good development prospect. Nevertheless, the development of any new method requires a long process and time period hence, multi-center, high-quality and prospective studies are required.
